# Supplementary material for: The Screening of the Protective Antigens of Aeromonas hydrophila Using the Reverse Vaccinology Approach: Potential Candidates for Subunit Vaccine Development
Source: Vaccines (Basel). 2023 Jul 21;11(7):1266. doi: 10.3390/vaccines11071266 (PMC10383140; doi:10.3390/vaccines11071266)
Supplement: Supplementary file 1 [file vaccines-11-01266-s001.zip › vaccines-2481916-Tables S1 and S2.pdf]

vaccines

MDPI

Supplemental material

Table S1. The sequence of the primers.

Protein	Name	Sequences (5'-3')
AJE34457.1	1-CDS-F	GATAAACAGAAAATTGCGTAGCGG
	1-CDS-R	ATTCGTCGTCATCAAATAACCCTT
AJE34471.1	2-CDS-F	CTTAATAGGGAATCCGGTGAAAAT
	2-CDS-R	GCCTCGCTTTTTATCTTCTCTTAT
AJE34515.1	3-CDS-F	CAACAGATGGGATAACCTGATGGA
	3-CDS-R	AACACCGATTGGCTATATCAGAGG
AJE34700.1	4-CDS-F	ATCAGCTGATGTATGGCGCCAAAT
	4-CDS-R	ACCTTTTCTGGTCCTGGTTTCACG
AJE34708.1	5-CDS-F	AACTCCGCATTCACATCCCTTTAT
	5-CDS-R	GAGGGGAGACCGAGGGTATTTTAT
AJE35401.1	6-CDS-F	CTGACACTTATGTGGATTTGATTG
	6-CDS-R	TCAATAGAAATCAGGACTGGCAAC
AJE35595.1	7-CDS-F	CTATGCTCTCGTCGTCGATAACAA
	7-CDS-R	CTCTGTTCTGTTGACTGGCATTAT
AJE35662.1	8-CDS-F	TATTTCACCAAGCACAGCACCAAG
	8-CDS-R	ACGGTAAGATAACGCAAGGAGAGA
AJE35814.1	9-CDS-F	CTGGTGGGTTTGACAATAAAGAGG
	9-CDS-R	CATAACCAGATCCACGGGACCAAC
AJE35877.1	10-CDS-F	CTGAGAGAACGATAAGCCAACAAT
	10-CDS-R	TACTGCTCAGTCACATGCATCTTC
AJE36112.1	11-CDS-F	CAGTGAGAGACAGATGAGCAGGAA
	11-CDS-R	GGAATAACAATAACCCCGAGTCTG
AJE36246.1	12-CDS-F	CAGAAACGGTCAATAACTGGCATA
	12-CDS-R	TATTAGCAGCACATCACCCAGCAT
AJE36247.1	13-CDS-F	AAATGCTGGGTGATGTGCTGCTAA
	13-CDS-R	ATCTGCCTACAAGAGAGAACACTA
AJE36909.1	14-CDS-F	TCCCCCCTTTCATCCCCTGTCTGC
	14-CDS-R	TGAGGAATAAGCGGGGCCACGATC
AJE37341.1	15-CDS-F	TGCCATCTCAACGAGGATTTTTTT
	15-CDS-R	GAAACGATTTGACAGAAAGTGACG
AJE37342.1	16-CDS-F	GAGTATCCAGGAACCATCTCTTTT
	16-CDS-R	CCTCGTCGATGTCTGGTGTAGTAG
AJE37343.1	17-CDS-F	CCTACTACACCAGACATCGACGAG
	17-CDS-R	CTTGCTCACCACCTGTTGTTATCT
AJE37434.1	18-CDS-F	TCACACTTACTCCTTGTTCCCTCA
	18-CDS-R	CAGAGCATCAACAAGGACACATAA
AJE37449.1	19-CDS-F	CGATAGACATCTTTACCAGTTTCA
	19-CDS-R	TATTTCGGTTGATTGTCATCGTAC
AJE37487.1	20-CDS-F	GCAATCGGATAAATAGCAAGCGTT
	20-CDS-R	AGAAGCGTGATGATCTGCAGGAAA
AJE37516.1	21-CDS-F	ATAACTATTGCGGACTGATACGGC
	21-CDS-R	TTCGTGGAGCCTGTCTTGATTGTT
AJE37629.1	22-CDS-F	CCTCCTCCTGGTTAGTGTTGCAAT
	22-CDS-R	AAACAGAGGGGCGTAGTGTAGAAC
AJE37705.1	23-CDS-F	CTCGATGAATCCTGTCGTAAAATG
	23-CDS-R	CAACGTCTTCTTCATATCCATCTC
AJE37778.1	24-CDS-F	GCTTCAGTTTCTATCAAGGTTCGC
	24-CDS-R	TCAACATCTGACGGGCAATAAAAA
AJE37804.1	25-CDS-F	GGCGTCCATCACCTTGTTCAGTTC
	25-CDS-R	TGCTGGGTTGGCTCTGTATTGGTT
AJE38163.1	26-CDS-F	CGGCCCTTTCACCAATTTGACATC
	26-CDS-R	ATCTCCTCGTCGTGGTTCTGTTGA
AJE38201.1	27-CDS-F	CTTGCCATTGTCGTAAGCAGTTTG
	27-CDS-R	GGATGGAGATGTGTGTGCCGATAG

AJE38225.1	28-CDS-F	AGAGCCCACACAGGAAAGACAACA
	28-CDS-R	GATGAGGCCAGTAGGGTGAGGTTG
AJE38562.1	29-CDS-F	GGGCACCAATAATAAGAAATCAGA
	29-CDS-R	AAACCAGAGACAAGAGTTCCATAA
AJE34456.1	30-CDS-F	CAGAGCCGTCAGCAGAGTCAAATT
	30-CDS-R	AGTGGAAACTGGTATGTTCGCAAG
AJE34989.1	31-CDS-F	TTCCCGTCGCACTGGGTAAAATGA
	31-CDS-R	ATGGGGACATCGTCATCAGCAGTT
AJE35148.1	32-CDS-F	AAACCCTTTCTGAACGGATGTGTC
	32-CDS-R	CAACAAGCCGTTTGAAATCAGCAG
AJE37118.1	33-CDS-F	GCCTGAAATTAAACAGCGGAAACA
	33-CDS-R	GACCATAGGGACAATAGAGCCACT
AJE37120.1	34-CDS-F	GCCAAGGGAGTAGGTATGATTCGG
	34-CDS-R	CTGCTCTCCCTGTGTGATGAAAGT
AJE37322.1	35-CDS-F	TAACAAACAAGTACCGCTATCCCT
	35-CDS-R	TTATTGTCAGTCCTTGTTTTGGGT
AJE37560.1	36-CDS-F	GTCCATAAAATCAGGAGCAATACA
	36-CDS-R	GTTTGCTAACTTCAACCACCTGCT
AJE37735.1	37-CDS-F	CTCCCCGATATTCGCCGTACAGAA
	37-CDS-R	TGCTCGACTCGCTCGGGATCTGAT
AJE37742.1	38-CDS-F	GCAAATCGTCGTTCACCGCCAATA
	38-CDS-R	TTCTGCGAACCGAGTCCTATCCCG
AJE38071.1	39-CDS-F	GCGTAGAGTTGAATCACTGACCGA
	39-CDS-R	CAGCACCCTGGTTACGCCATCTTC

Note: Starting from the CDS region of the 39 proteins, 210 bases were counted both forward and backward. Primers for 39 proteins were designed using Primer Premier 5.0 to ensure amplification of the intact CDS region.

Table S2. 24 strains of *Aeromonas* sp. were selected from the NCBI NR database.

Species	Strain Name	Accession number
A. caviae	GSH8M-1	AP019195.1
	R25-6 chromosome	CP025705.1
	WP8-S17-ESBL-03	AP022214.1
	CYEY0630 chromosome	CP118442.1
	Aero52 chromosome	CP066813.1
	BC02 chromosome	CP102320.1
A. salmonicida	A449	CP000644.1
	O23A chromosome	CP021654.1
	A527 chromosome	CP022550.1
	S44	CP022181.1
	AS2 chromosome	CP110652.1
	FN1	CP101948.1
A. veronii	*WP2-S18-CRE-03*	*AP021940.1*
	B565	CP002607.1
	SW3814 chromosome	CP083461.2
	TH0426	CP012504.1
	AVNIH1	CP014774.1
	CB51 chromosome	CP015448.1
A. hydrophila	KAM330	AP023398.1
	ML09-119	CP005966.1
	NJ-35	CP006870.1
	YL17	CP007518.2
	pc104A	CP007576.1
	AL09-71	CP007566.1

Note: Twenty-four strains of *Aeromonas* species were selected from the NR database of NCBI.
